# Urinary Metabolic Phenotyping Reveals Differences in the Metabolic Status of Healthy and Inflammatory Bowel Disease (IBD) Children in Relation to Growth and Disease Activity

**DOI:** 10.3390/ijms17081310

**Published:** 2016-08-11

**Authors:** Francois-Pierre Martin, Jessica Ezri, Ornella Cominetti, Laeticia Da Silva, Martin Kussmann, Jean-Philippe Godin, Andreas Nydegger

**Affiliations:** 1 Nestlé Institute of Health Sciences, 1015 Lausanne, Switzerland; ornella.cominetti@rd.nestle.com (O.C.); Laeticia.DaSilva@rd.nestle.com (L.D.S.); Martin.Kussmann@rd.nestle.com (M.K.); 2 Division of Pediatric Gastroenterology, University of Lausanne, 1011 Lausanne, Switzerland; jessica.ezri@chuv.ch; 3 Nestlé Research Center, 1000 Lausanne, Switzerland; jean-philippe.godin@rdls.nestle.com

**Keywords:** pediatric, metabolism, phenotype, growth, inflammatory bowel disease, Crohn’s disease, ulcerative colitis

## Abstract

Background: Growth failure and delayed puberty are well known features of children and adolescents with inflammatory bowel disease (IBD), in addition to the chronic course of the disease. Urinary metabonomics was applied in order to better understand metabolic changes between healthy and IBD children. Methods: 21 Pediatric patients with IBD (mean age 14.8 years, 8 males) were enrolled from the Pediatric Gastroenterology Outpatient Clinic over two years. Clinical and biological data were collected at baseline, 6, and 12 months. 27 healthy children (mean age 12.9 years, 16 males) were assessed at baseline. Urine samples were collected at each visit and subjected to ^1^H Nuclear Magnetic Resonance (NMR) spectroscopy. Results: Using ^1^H NMR metabonomics, we determined that urine metabolic profiles of IBD children differ significantly from healthy controls. Metabolic differences include central energy metabolism, amino acid, and gut microbial metabolic pathways. The analysis described that combined urinary urea and phenylacetylglutamine—two readouts of nitrogen metabolism—may be relevant to monitor metabolic status in the course of disease. Conclusion: Non-invasive sampling of urine followed by metabonomic profiling can elucidate and monitor the metabolic status of children in relation to disease status. Further developments of omic-approaches in pediatric research might deliver novel nutritional and metabolic hypotheses.

## 1. Introduction

Whilst the prevalence of inflammatory bowel disease (IBD) has increased considerably over recent decades, its clinical features do not allow accurate prediction of disease progression or response to therapy [1]. Approximately a quarter of patients will develop IBD during childhood and adolescence, the majority of them around their pubertal growth spurt [2]. Growth failure and delayed puberty are major complications in pediatric patients with IBD, especially in those with Crohn’s disease (CD) [2,3]. These features are already present before the onset of clinical symptoms, with a frequency ranging from 14% to 88% of patients [4]. Therefore, optimization of growth is one of the critical aims in the management of pediatric IBD. However, growth delay might persist despite reduced disease activity [5,6], with diminished final adult height in almost one in five IBD children [7]. The origin of growth retardation is multifactorial, including malnutrition, active inflammation, and steroid therapy among the principal determinants [2,8,9]. Malnutrition is mainly due to anorexia induced by inflammation [10,11], reduced energy intake due to digestive symptoms, and malabsorption of nutrients. Furthermore, CD children with growth failure have normal growth hormone (GH) secretion but diminished plasma concentration of insulin-like growth factor-1 (IGF-1), suggesting a certain degree of GH resistance that may be related to malnutrition and inflammation [12].

Metabonomics is nowadays commonly used as a systems biology approach to explore physiological regulatory processes in human clinical research with regard to disease etiology, diagnostic stratification and potentially to mechanisms of action of therapeutic solutions. Metabonomics has been defined as the quantitative measurement of dynamic metabolic changes of living systems in response to genetic modifications or physiological stimuli, including nutrients and drugs [13,14]. Metabonomics is achieved through global or targeted profiling of low molecular weight molecules in biofluids, as diverse as blood, urine, saliva, cerebrospinal fluid, as well as in stools and tissues [15]. Since the measured biochemical species are the products and by-products of the various biochemical pathways existing in all living systems, metabonomics is a well-established approach to capture and monitor intra- and extra-cellular regulatory processes [16]. From an analytical approach, metabonomics is based on either proton nuclear magnetic resonance (^1^H NMR) spectroscopy or mass spectrometry (MS). MS methods can be combined with a separation of metabolites using either gas chromatography or liquid chromatography. Both NMR and MS methods generate high density data, from which meaningful biological information are recovered using multivariate data analytical approaches [17,18]. Metabonomics has already begun to contribute to the field by generating key metabolic insights [1,19,20,21]. In the context of the study of pediatric subjects, metabonomics offers a unique opportunity to capture metabolic fingerprints of an individual using minimally invasive samples, such as blood spots or urine. Application of metabonomics to urine is a robust approach to generate metabolic phenotypes that associate with time-averaged representations of recent biochemical events within an organism, including gut microbial metabolic interactions with host metabolic pathways [22,23].

In this study, we applied ^1^H NMR-based metabonomics to characterize the biochemical fingerprints of urine samples from IBD and healthy patients. Advanced clinical and anthropometric phenotyping were also conducted on IBD children, where association with their metabolic status was explored to identify biochemical processes varying according to growth and disease activity (over one year with three visits at six-month intervals).

## 2. Results

### 2.1. Clinical Parameters of IBD and Healthy Subjects

A total of 21 (15 Crohn’s disease, CD, 6 ulcerative colitis, UC) pediatric patients and 27 healthy children were enrolled during the study period, with urine samples available for metabonomics analysis and extensive clinical phenotyping. The IBD population was chronologically slightly older than the healthy group (Table 1). Moreover, CD patients showed lower z-scores for body weight (*p* < 0.01), height (*p* < 0.01) and body mass index (BMI) (*p* < 0.05) at baseline and throughout the follow-up of the IBD patients (Table 1). At baseline, CD patients had lower resting energy expenditure (*p* < 0.05). No significant differences were noted overtime in the biological parameters in IBD patients (Table 1). 

### 2.2. Urine Metabonomics Describes Differences between IBD and Healthy Subjects

For metabonomics urinary analysis, two metabolic profiles were discarded due to extreme dilution (one from a healthy subject at baseline, and one from a CD subject at the six-month visit). Multivariate data analysis was performed on the metabolic profiles using principal component analysis (PCA) and a modification of Projection to Latent Structures Discriminant Analysis (OPLS-DA). These multivariate data analyses explore the variance in the metabonomics that may explain statistical differences between groups of samples. Here, significant metabolite concentration differences were observed in the urine composition between IBD patients and healthy subjects at baseline, as noted by the OPLS-DA model generated with one predictive and two orthogonal components (*R*^2^*X* = 0.17, *R*^2^*Y* = 0.96, *Q*^2^*Y* = 0.18, where *R*^2^*X* corresponds to the explained variance in the metabonomics data (urine metabolites), *R*^2^*Y* to the explained group variance (healthy and IBD groups) and *Q*^2^*Y* to the robustness of the model). 

**Figure 1:**
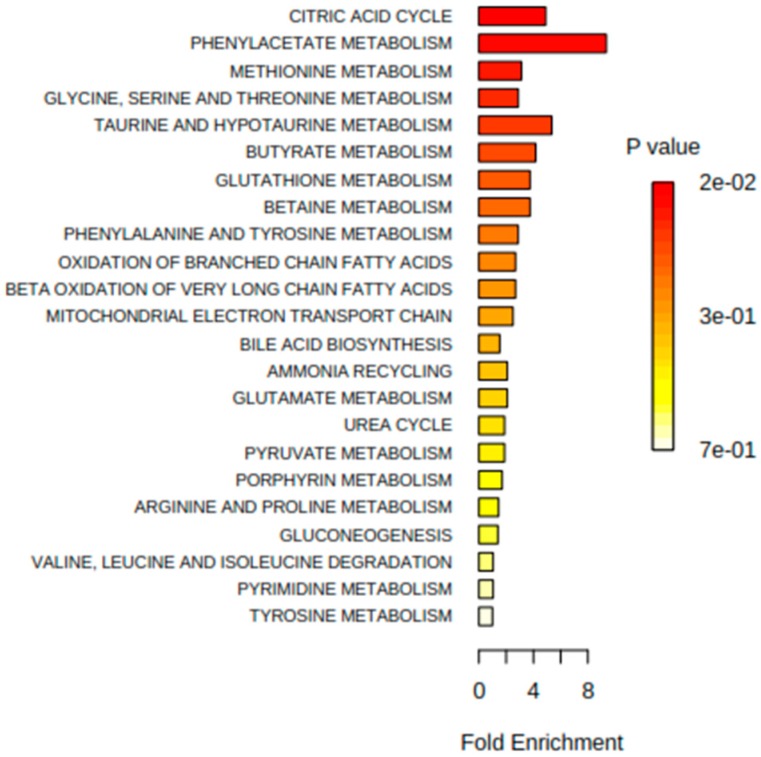
Summary plot of over representation analysis of urinary metabolites, using metabolite set enrichment analysis (MSEA).

Additional analyses highlighted that the metabolic differences between IBD and healthy children were also present after 6 months (*R*^2^*X* = 0.20, *R*^2^*Y* = 0.94, *Q*^2^*Y* = 0.13) and 12 months of monitoring (*R*^2^*X* = 0.20, *R*^2^*Y* = 0.86, *Q*^2^*Y* = 0.24). Inspection of the model loadings allowed the identification of influential metabolites contributing to discriminate the groups of subjects by multivariate analysis. Representative signals of metabolites were integrated and reported in Table 2. Metabolite set enrichment analysis (MSEA) [24] was applied to identify meaningful patterns that are significantly enriched on the quantitative metabonomics data. Briefly, MSEA enables the identification of small but consistent changes among a group of related compounds. Here, major metabolic changes related to IBD conditions pointed towards the Krebs cycle and amino acid metabolic pathways (Figure 1).

Variables identified by multivariate analysis were further probed by univariate testing as indicated in Table 2, with the aim to describe variations in urinary excretion of these metabolites. When compared to healthy subjects, pediatric IBD patients show higher urinary excretion of phenylacetylglutamine (PAG, *p* < 0.05), and lower urinary excretion of *cis*-aconitate (*p* < 0.05), hippurate (*p* < 0.05), and urea (*p* < 0.05). Additional inspection of data showed sub-group specificities (see Supplementary Materials). In particular, CD subjects were characterized with a lower excretion of carnitine when compared to healthy subjects.

### 2.3. Integration of Clinical and Urine Metabonomics Data in IBD Patients

**Figure 2:**
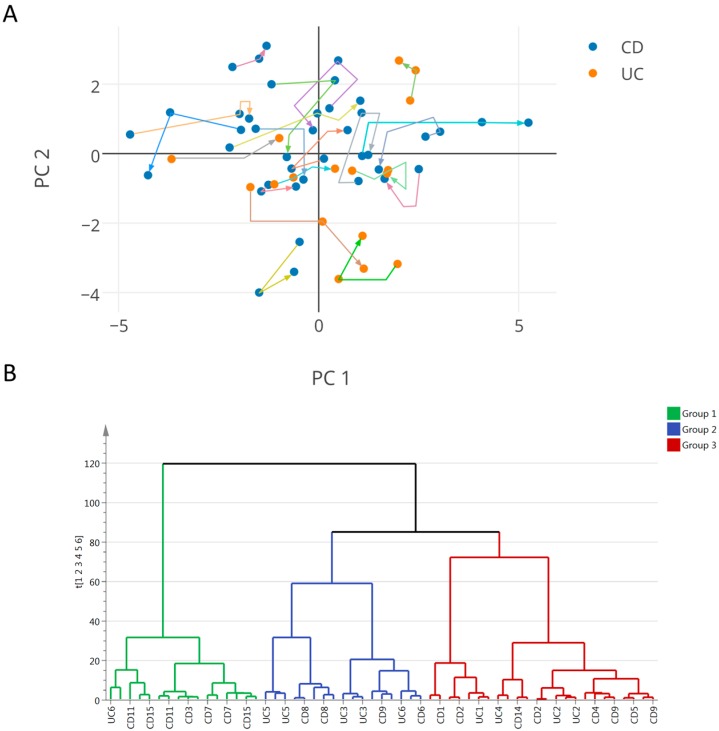
(**A**) Principal component analyses of clinical and biological parameters in inflammatory bowel disease (IBD) over time. Data points in orange represent samples (at either of the three time points) of ulcerative colitis (UC) patients and in blue, samples of Crohn’s disease (CD) patients. Trajectories of individual subjects are depicted in unique colors, with the arrows indicating the directions of the time points; (**B**) Dendrogram obtained by performing hierarchical clustering analysis (HCA) (average linkage) showing three groups of patients of similar size, which may be further subdivided into smaller clusters. By visual inspection of the tree, three groups were obtained by cutting the tree at the top nodes which relates to different clinical status.

In this exploratory study, the analysis aims for the identification of shared metabolic features linked to changes in growth and disease activity, irrespectively of the therapeutic treatment. Analysis of variance using principal component analysis (PCA) was applied only on clinical and anthropometric data of IBD patients (%FFM, Tanner score, age, height z-score, weight z-score, BMI z-score, growth velocity z-score, fecal calprotectin, erythrocyte sedimentation rate (ESR), CRP, urea, IGF-1, insulin-like growth factor-binding protein 3 (IGFBP-3), caloric intake, and resting energy expenditure (REE)). An overall PCA model was generated with six principal components (PCs) explaining 81% of the total variance of the data, the first two components explaining 44% of the variance. Data were visualized by means of principal component scores (Figure 2A), where each point represents an individual at a given time point based on its clinical and anthropometric data. The clinical variables responsible for any detected differences between samples in the scores plot can be extracted from the corresponding loadings plot, where each coordinate represents a single clinical parameter. The distribution of the patients along the first component was driven by the values for %FFM and REE for which variations were negatively associated with fecal calprotectin values (i.e., a subject with low %FFM, REE values has high calprotectin). Along the second component, the distribution of the subjects was determined by variations associated to caloric intake, BMI-z score, weight-z score, ESR, and fecal calprotectin. The plotting of individual trajectories in this PCA space (Figure 2A) showed that some subjects have clinical sub-phenotypes evolving over the duration of the study.

Based on the PCA scores, sub-groups of IBD patients were objectively defined in order to subsequently assess their distinctive clinical and metabolic status. To achieve this, hierarchical clustering analysis (HCA) was performed on the PCA scores generated previously, as illustrated in Figure 2B. The dendrogram obtained by performing HCA (average linkage) showed three clusters of patients of similar size. These groups were defined by visual inspection of the tree, the top nodes of which relate to different clinical statuses. For each defined group, clinical and biological parameters are reported in Table 3. In particular, group 1 was characterized by lower weight z-score, height z-score, BMI z-score, growth velocity z-score, %FFM, REE. Group 2 was characterized by a higher weight z-score and BMI z-score, lower urea, and lower caloric intake, whereas group 3 showed higher growth velocity z-score, and lower ESR and CRP.

Using these three groups defined according to clinical and anthropometric data, urine metabolic profiles were analyzed using supervised multivariate data analysis. A first OPLS-DA model showed the occurrence of statistically significant differences in the biochemical composition of urine between the three groups, as noted by the model parameters (*R*^2^*X* = 0.19, *R*^2^*Y* = 0.68, *Q*^2^*Y* = 0.25, 2 predictive and 1 orthogonal components). Additional pairwise comparisons highlighted group-specific signatures using one predictive and one orthogonal component; group 1 vs. group 2 (*R*^2^*X* = 0.15, *R*^2^*Y* = 0.91, *Q*^2^*Y* = 0.27), group 1 vs. 3 (*R*^2^*X* = 0.12, *R*^2^*Y* = 0.94, *Q*^2^*Y* = 0.44), and group 2 vs. group 3 (*R*^2^*X* = 0.13, *R*^2^*Y* = 0.90, *Q*^2^*Y* = 0.24). Inspection of the model loadings enabled the identification of variables contributing to the distinction of the groups of samples. Representative signals of the previously identified metabolites were integrated and tested by univariate analysis (Table 4).

Group 1 showed a distinctive urine profile, marked by a higher urine concentration of acylcarnitine compared to other IBD groups and healthy subjects. Group 2 showed higher levels of mannitol and an unassigned metabolite Uk3 when compared to the other IBD groups and healthy subjects, and the greatest increase in 4-hydroxyphenylacetate when compared to healthy subjects. Group 3 showed a low level of methanol compared to other IBD groups and healthy subjects, and had the greatest decrease in urinary urea when compared to healthy subjects. Furthermore, groups 2 and 3 showed high levels of 4-hydroxyphenylpyruvate, PAG, and tryptophan when compared to healthy subjects. 

In addition, to unravel more specific associations between urinary metabolites and clinical endpoints in CD patients, Spearman’s rank correlation analysis was performed and reported in Supplementary Figure S1. In particular, blood CRP and fecal calprotectin both showed negative correlations with urinary levels of PAG, 4-hydroxyphenylacetate, tryptophan, creatinine, as well as %FFM, height and growth velocity z-scores, and REE—but a positive correlation with urinary urea and formate. ESR showed positive correlations with the urinary level of formate and fecal calprotecin, but negative correlations with urinary levels of 4-hydroxyphenylpyruvate, *cis*-aconitate, 3-hydroxy-isobutyrate, and 3-methyl-2-oxovalerate, as well as blood IGFBP-3. 

## 3. Discussion

To the best of our knowledge, this is the first study showing a relation between clinical characteristics of pediatric patients with IBD and their urinary metabolic profiles over time in relation to disease state. Despite the limited number of subjects, the longitudinal experimental design with a healthy reference group offers key opportunities to explore metabolic status in childhood in relation to growth and disease state. Urinary metabolic profiles of IBD children differ significantly from healthy controls. Such metabolic differences include central energy metabolism (Krebs cycle), amino acid and gut microbial metabolic pathways, which are discussed here below.

### 3.1. Urine Metabonomics Reflects Different Metabolic Requirements in Pediatric IBD Patients Compared to Healthy Subjects

Generally, the pediatric IBD condition shows growth failure and weight loss as hallmarks of subjects with CD, but less with UC [8,25]. In our study, CD pediatric patients showed lower z-scores for body weight, body height, and BMI compared to healthy controls. Healthy controls generally had higher IGF-1 and IGFBP-3 levels—more likely through adequate secretion—with inferred effects on growth, muscle, and fat mass development. The concomitant decrease in resting energy expenditure for CD patients reveals further differences in whole-body energy metabolism and related metabolic processes [26,27]. Such hypotheses are supported by the urine analysis that shows variations in key metabolic pathways indicating changes in protein and energy metabolism. Such changes are described through differences in the urea and Krebs cycle, namely with a decreased urinary excretion of urea and *cis*-aconitate—a precursor of alpha-ketoglutarate. The urinary excretion pattern of phenylacetylglutamine (PAG), which is a major nitrogenous metabolite, was also found to be higher in IBD pediatric subjects. PAG synthesis depends on the availability of phenylacetate—from either host or gut-microbial metabolism—and glutamine, mainly generated in the Krebs cycle from alpha-ketoglutarate. PAG is a key means to shuttle excess nitrogen out of the body. Its increased urinary excretion closely mirrors the decreased levels of urinary urea and *cis*-aconitate. Interestingly, previous reports documented how PAG may also replace urea as a waste of nitrogen product in specific disease conditions, such as in uremic patients [28,29,30]. In contrast to studies with adult IBD patients [31,32,33], fasting blood urea, urine citrate, and succinate remained unchanged in this pediatric cohort. This peculiar excretion of end products of protein metabolism indicates a different handling of nitrogen in pediatric IBD patients.

### 3.2. IBD Clinical Sub-Phenotypes Link to Different Metabolic Status

Based on the IBD population characteristics, three clinical sub-phenotypes could be ascribed. Group 1 was characterized by lower weight z-score, height z-score, BMI z-score, growth velocity z-score, %FFM, and REE. This cluster corresponds to the pediatric patients with chronic mild disease state. Group 2 was characterized by a higher weight z-score and BMI z-score, lower urea, lower caloric intake, whereas group 3 showed higher growth velocity z-score, lower ESR and CRP. This set of clinical endpoints might be seen as two different stages of patients with growth improvement with group 2 reflecting longstanding remission or diseases interfering less with growth (i.e., UC), and group 3 showing patients with catch-up growth.

Metabonomics analysis has been able to ascribe specific metabolites to disease sub-phenotypes. For instance, groups 2 and 3 correspond to patients having stable growth or growth improvement, as well as reduced inflammatory status. It is therefore worth noting that groups 2 and 3 have higher urinary levels of PAG and tryptophan, compared to healthy subjects. Supported by correlation analysis, this pattern suggests a metabolic relationship linking PAG and tryptophan to changes in %FFM, growth, and inflammatory conditions in pediatric patients in remission. Similarly, the reduced urinary excretion of methanol and urea in group 3, two metabolites significantly correlated to CRP and fecal calprotectin, may serve as readout for monitoring patients that recover towards remission and show growth improvement. Such observation also further supports the relevance of urinary urea and PAG for monitoring protein and muscle metabolism in pediatric patients with IBD.

Last but not least, the group 1 shows a distinctive urine profile marked by higher urinary excretion of acylcarnitine compared to other IBD groups and healthy subjects. Despite the fact that the urinary pattern did not correlate with any studied clinical endpoint, such metabolic readouts illustrate the need to further study the different metabolic requirements in fatty acid use and oxidation under inflamed conditions.

### 3.3. Host-Gut Microbial Urinary Co-Metabolites Describe Relationships between Dietary Sources of Nitrogen, Carbamyl Phosphate Synthetase, and Host Metabolism

As already reported in adults with IBD [31,32,33], the urinary excretion of hippurate was decreased in children with IBD compared to healthy controls. Williams et al. previously reported that dietary factors and deficit in the conjugation of benzoate to glycine did not explain the differences in the metabolism of hippurate [33], thus providing strong evidence for dysbiosis. Indeed, IBD is associated with reduced microbiota diversity, lower microbial capacity for butyrate production and increased pro-inflammatory bacteria [1,34], some features being further discussed in Supplementary Materials. Furthermore, despite urinary hippurate being significantly different from healthy subjects, our analysis did not show any differences amongst the IBD sub-phenotypes. In addition, groups 2 and 3 tended to have an increased urinary excretion of other gut microbial metabolites; 4-hydroxyphenylacetate and 4-hydroxyphenylpyruvate, as compared to healthy subjects. These differences also indicate persistent changes in microbial metabolism and processing of dietary components. Since these human-microbial metabolites correlate with inflammatory markers (CRP, ESR), their relevance to monitoring the normalization of gut microbial metabolic processes in pediatric IBD should be further investigated.

Previous relationships between urinary urea nitrogen excretion and appearance of urine hippurate and/or PAG nitrogen were reported in normal subjects given sodium benzoate or sodium phenylacetate, respectively [29]. It is also important to note that the use of amino acid acylation pathways has been successfully exploited in empiric studies of patients with inborn errors of urea synthesis (e.g., carbamyl phosphate synthetase (CAD) deficiency) [29]. In the management of such clinical conditions, treatment with sodium benzoate and sodium phenylacetate activates the synthesis and excretion of hippurate and PAG, both of which may serve as waste nitrogen products [29]. Moreover, we found that PAG is positively correlated to %FFM and urine creatinine, but negatively correlated to urinary urea and inflammatory markers CRP and calprotectin. This may be of particular importance, as monitoring nitrogen excretion gives insights into the state of growth of a subject in childhood and net degradation of protein. Our current study suggests that the increased excretion of nitrogen products is related to an increase in fat free mass in CD pediatric patients, and one of its other markers—creatinine [35]—in parallel to decreased inflammatory conditions. 

In the context of IBD, CAD, an enzyme required for de novo pyrimidine nucleotide synthesis; was identified as a NOD2-interacting protein expressed at increased levels in the colon epithelium of patients with CD compared with controls [36]. The bacterial sensor NOD2 has been associated with CD, and the authors speculate that CAD is a negative regulator of NOD2 and might be a pharmacologic target for CD therapies [36]. Therefore, the relationships between urinary nitrogen excretion through urea, hippurate, and PAG may be a potential readout for CAD-NOD2 activity in pediatric IBD. Moreover, as already reported in adults with IBD [31,32,33], the urinary excretion of hippurate was decreased in children with IBD as compared to healthy controls. The main source of variations in hippurate metabolism comes from dietary factors (e.g., dietary sources of polyphenolic compounds such as fruits and vegetables), and hepatic and gut microbial metabolism of its precursors (mainly benzoic acid) [37,38]. Williams et al. previously provided a strong evidence for dysbiosis [33]. In particular, IBD was associated with reduced microbiota diversity, lower microbial capacity for butyrate production and increased pro-inflammatory bacteria [1,34]. Furthermore, UC patients show a consistent trend towards higher levels of other gut microbial metabolites, 4-hydroxyphenylacetate and 4-hydroxyphenypyruvate, that are mainly formed in the colon by bacterial fermentation [38,39], which may support region-specificity of gut metabolic dysbiosis.

Patients with UC have a consistent trend in higher urinary excretion of two products of branched chain amino acid (BCAA) metabolism, 3-methyl-2-oxovalerate and 2-oxoisocaproate, and lactate—end product of anaerobic carbohydrate metabolism, suggesting an upregulation of BCAA and carbohydrate catabolism. Concomitantly, urinary excretion of fatty acid β-oxidation intermediates, carnitine and acylcarnitine, tends to decrease, thus indicating a downregulation of fatty acid breakdown through β-oxidation. Taken together with changes in PAG and the Krebs cycle, this urinary pattern describes a further remodeling of energy, amino acid and fatty acid metabolism in relation to the altered metabolic requirements of UC pediatric patients. 

## 4. Materials and Methods

### 4.1. Subjects

Eligible patients were aged between 10 and 18 years old, with a diagnosis of CD or UC, confirmed according to international criteria [40]. IBD subjects were assessed at baseline (T0), after 6 (T6) and 12 months (T12), respectively. All patients were in remission and underwent therapeutical management of the disease according to recommended drugs (see supplementary Table S1 for information). To be noted that none was treated with enteral nutrition and no endoscopy was performed to assess mucosal inflammation.

Control healthy subjects were recruited among the general pediatric population. They were matched for age, pubertal stage, and gender to the IBD subjects. They had neither chronic inflammatory disease nor family history of inflammatory bowel. Anthropometric data and urine samples for metabolic analyses were collected at one time point. 

An informed written consent was obtained from the parents and an oral assent from each child. 

### 4.2. Anthropometric and Clinical Measures

#### 4.2.1. Anthropometric Assessment 

Body weight was measured using a calibrated digital scale (Seca, Hamburg, Germany) to the nearest 0.1 kg. Height was measured using a wall-mounted stadiometer (Holtain, Crosswell, UK) to the nearest 0.1 cm. Body mass index (BMI, kg/m^2^) was determined by dividing the weight in kilograms by the square of the height in meters. Height velocity was calculated as the amount of growth in centimeters divided by the time interval between measurements in years. All values were expressed in z-scores [41,42]. Pubertal stage was assessed according to Tanner score [43]. 

#### 4.2.2. Body Composition 

Bioimpedance analysis (BIA) was performed using Body Impedance Analyzer Akern (Florence, Italy). While the subject was lying comfortably without his limbs touching the body, electrodes were placed just below the phalangeal-metacarpal arch in the middle of the dorsal side of the dominant hand and just below the metatarsal arch on the superior side of the foot of the same side. Fat free mass in kg (FFM) was then calculated using the software BodyGram Pro^®^ supplied by the manufacturer (which uses weight, age, and an impedance index (height^2^/resistance)) [44,45]. Percentage of FFM (%FFM) was calculated by dividing FFM with the body weight of the subject expressed in kg.

#### 4.2.3. Disease Activity in Patients with IBD

Disease activity was scored using the pediatric Crohn’s disease activity index (PCDAI) [46] for CD, a 100 point scale where a score >30 indicates severe disease, and the pediatric ulcerative colitis activity index (PUCAI) [47] for UC, an 85-point scale where a score >35 indicates severe disease. Remission was defined as PCDAI or PUCAI score lower than 10. No endoscopic control was performed since all patients were in remission.

#### 4.2.4. Blood and Stool Markers

Inflammatory markers (erythrocyte sedimentation rate (ESR), C-reactive protein (CRP)), urea, and growth factors (insulin-like growth factor 1 (IGF-1) and insulin-like growth factor-binding protein 3 (IGFBP-3), expressed in z-scores) were obtained after a fasting period of at least 6 h. Fecal calprotectin was measured and a cut-off value of 275 μg/g was set to determine possible relapse of disease [48].

#### 4.2.5. Dietary Intake

All subjects underwent a 24-h food recall with the help of a questionnaire showing pictures of different sizes of plates for the different foods with the same examiner (dietician). Qualitative and quantitative analyses were made using the software Prodi 5.8 Expert (Nutri-Science GmbH, Hausach, Deutschland, Germany). Daily intake was expressed as kcal per day.

#### 4.2.6. Resting Energy Expenditure

Resting energy expenditure (REE, kcal) was measured using Quark RMR (Cosmed, pulmonary function equipment, Delta Medical, Rome, Italy). Prior to each measurement, the indirect calorimeter was calibrated with a standard gas of a known composition (95% O_2_, 5% CO_2_). Measurements were performed in a quiet thermoneutral room (20 °C) after a fasting period of at least 6 h, to minimize any effect attributable to the thermic effect of food. Oxygen consumption and carbon dioxide production were measured every 5 s for at least 20 min and REE was defined as the mean energy expenditure over the measured period.

### 4.3. Metabonomics Analysis

Morning spot urine samples were collected at baseline for all subjects, and at the 6-month and 12-month visit for the IBD patients. Urine samples (1 mL) were collected by means of sterile plastic tubes, and were stored at −80 °C, prior to analysis. 40 µL of urine were mixed with 20 µL deuterated phosphate buffer solution 0.6 M KH_2_PO_4_, containing 1 mM of sodium 3-(trimethylsilyl)-[2,2,3,3-2H_4_]-1-propionate (TSP, chemical shift reference *δ*_H_ = 0.0 ppm). The homogenates were centrifuged at 17,000× *g* for 10 min and 60 µL of the supernatant were transferred into 1.7 mm NMR tubes. ^1^H NMR metabolic profiles were acquired with a BrukerAvance II 600 MHz spectrometer equipped with a 1.7 mm probehead 300 K (BrukerBiospin, Rheinstetten, Germany), using a standard pulse sequence with water suppression, and processed using TOPSPIN (version 2.1, Bruker, Germany) software package.

### 4.4. Statistical Analysis

Chemometric analysis was performed on clinical and metabonomics data using the software package SIMCA-P+ (version 12.0, Umetrics AB, Umeå, Sweden). Principal component analysis (PCA) and a modification of partial least squares regression (PLSR) that removes all information orthogonal to the response variable during the fitting process were employed. This variant, orthogonal projection to latent structures (O-PLS) [49] provides sparser models (improving their interpretability) with the same degree of fit as PLSR. To highlight the weight of individual variables in the model, variable importance in projection (VIP) was used, with a value above 1 used as a threshold by convention. Influential metabolites were relatively quantified by signal integration and analyzed using *t*-tests. Metabolic pathway analysis was conducted by performing a metabolite set enrichment analysis, using the web-based MetaboAnalyst 3.0 tool [24], to the list of influential metabolites obtained through multivariate data analysis. Visualization of the trajectories in the principal components (PC) space was performed using Plotly (Plotfly Technologies Inc., Montréal, QC, Canada).

### 4.5. Ethics

This clinical study was approved by the Ethical Committee of the University of Lausanne, Switzerland (protocol 69/10) on 22 March 2010, and conducted in the Pediatric Gastroenterology outpatient clinic of the University Hospital of Lausanne, Switzerland. Informed written consent was obtained from the patients and their parents. 

## 5. Conclusions 

The present study shows how non-invasive sampling of urine followed by metabonomic analysis might elucidate and monitor the metabolic status of children in relation to disease state. Such metabolic profiles provide biological insights into host and bacterial metabolism by means of which we might assess metabolic fingerprints at different stages of disease. Despite the limited number of subjects, the longitudinal experimental design enabled the identification of a peculiar metabolite pattern to monitor metabolic requirements. Urinary urea and phenylacetylglutamine—two readouts of nitrogen metabolism—appeared particularly relevant and should be further investigated in follow-up studies. In particular, the levels of these particular metabolites correlate with the level of FFM in pediatric subjects, and could offer cost-effective alternative to DXA or bioelectrical impedance analysis, and enable regular assessment of lean mass for optimal growth catch-up under standard care practice. Therefore, further developments of such omic-approaches in pediatric research are needed and will deliver novel nutritional and metabolic hypotheses.

## Figures and Tables

**Table 1:** Population characteristics.

Group	Healthy Controls	Crohn’s Disease (CD)			Ulcerative Colitis (UC)		
Visit	T0	T0	T6	T12	T0	T6	T12
N total (males)	27 (16)	15 (8)	14 (8)	12 (7)	5 * (2)	6 (2)	6 (2)
Age (Years)	12.9 ± 1.9 (10.1–16.7)	14.9 ± 1.3 ^b^ (12.4–16.7)	15.2 ± 1.2 (12.9–17.2)	15.7 ± 1.3 (13.4–17.7)	15 ± 1.2 ^a^ (12.7–16.1)	15.2 ± 1.3 (13.1–16.7)	15.8 ± 1.2 (13.8–17.2)
Tanner Score	3 ± 1 (1–5)	3 ± 1 (2–5)	4 ± 1 (2–5)	4 ± 1(2–5)	4 ± 1 (3–5)	4 ± 1 (2–5)	4 ± 1 (3–5)
Weight z-score	0.5 ± 1 (−2.1–1.8)	−0.7 ± 0.9 ^b^ (−2.1–1.2)	−0.7 ± 0.8 (−1.8–0.9)	−0.7 ± 0.9 (−2.2–0.6)	−0.1 ± 0.9 (−1.3–1.2)	−0.3 ± 1 (−1.5–0.9)	−0.1 ± 1 (−1.6–1)
Height z-score	0.5 ± 0.9 (−1.7–2.2)	−0.8 ± 1 ^b^ (−3.2–0.5)	−0.7 ± 1 (−3.1–1.1)	−0.5 ± 1.1 (−2.5–1.7)	−0.1 ± 1.5 (−2.4–1.8)	−0.3 ± 1.2 (−2.1–1.6)	−0.2 ± 1.1 (−1.8–1.5)
BMI z-score	0.4 ± 0.9 (−1.7–1.6)	−0.5 ± 1.2 ^a^ (−2.8–1.7)	−0.5 ± 1 (−2.3–1.4)	−0.7 ± 1.1 (−2.7–1.1)	0 ± 0.9 (−1.1–1.1)	−0.2 ± 1 (−1.3–1.2)	−0.1 ± 1 (−1.3–1.6)
GV z-score	NA	0.8 ± 1.7 (−1.4–3.8)	0.7 ± 1.6 (−1.1–4.3)	0.9 ± 1.7 (−1–4.6)	0.1 ± 0.6 (−0.4–1.4)	−0.3 ± 1.4 (−1.8–2.1)	0.4 ± 1.2 (−0.8–2.7)
%FFM	39.6 ± 10.9 (23.5–60.6)	35.0 ± 5.1 (26.7–43.7)	37.3 ± 5.4 (27.7–48.1)	38.9 ± 7.3 (29.7–57.7)	37.8 ± 4.1 (33.4–43.8)	36.7 ± 4.2 (27.8–40.2)	39.4 ± 5.1 (29.6–44.8)
REE (Kcal)	1531.2 ± 275.6 (958–2036)	1338.1 ± 147 ^a^ (1065–1702)	1381.3 ± 188.6 (1050–1824)	1374 ± 213.2 (1177–1942)	1472.6 ± 86.6 (1362–1561)	1355.3 ± 201.8 (1118–1599)	1467.7 ± 174.6 (1249–1761)
Blood Urea (mmol/L)	438.8 ± 109.9 (116.8–647.9)	379.7 ± 111.2 (208.5–548.4)	395.9 ± 101.2 (224.4–541.5)	401.3 ± 123.4 (189.8–615.6)	394.4 ± 86 (310.7–545.7)	382.1 ± 145.1 (156.1–586.4)	389.5 ± 182.1 (90.7–598.6)
PCDAI in CD/PUCAI in UC	NA	9.8 ± 9 (0-30)	7.7 ± 7 (0-22.5)	6.3 ± 8.9 (0-25)	5 ± 4.5 (0-10)	5.8 ± 5.3 (0-15)	3 ± 4 (0-10)
ESR (mm/h)	NA	15.1 ± 8.5 (2–32)	16 ± 17.8 (3–70)	18 ± 20.2 (1–70)	26.6 ± 25.9 (9–78)	33.2 ± 31 (10–94)	23.8 ± 12.2 (13–47)
CRP ** (mg/L)	NA	3.5 ± 1.9 (2–8)	7.1 ± 10.1 (2–35)	11.1 ± 17.3 (2–60)	5 ± 3.3 (2–11)	6.5 ± 6.5 (2–18)	7.7 ± 11.4 (1–33)
Fecal calprotectin (µg/g)	NA	660.8 ± 673.9 (10–1500)	372.3 ± 464.4 (20–1500)	714.8 ± 643 (20–1500)	1046.7 ± 501.5 (367–1500)	966.7 ± 644.2 (20–1500)	1500 ± 0 (1500–1500)
IGF-1 z-score	NA	−0.6 ± 0.4 (−1.1–0.3)	−0.7 ± 0.3 (−1.1–0.0)	−0.7 ± 0.3 (−1.2–0.1)	−0.5 ± 0.5 (−1.0–0.1)	−0.4 ± 0.5 (−0.9–0.4)	−0.4 ± 0.4 (−0.9–0.4)
IGFBP-3 z-score	NA	−0.5 ± 0.2 (−0.9–−0.2)	−0.5 ± 0.1 (−0.8–−0.3)	−0.5 ± 0.2 (−0.7–0.1)	−0.4 ± 0.5 (−1.0–0.0)	−0.4 ± 0.4 (−0.8–0.1)	−0.4 ± 0.3 (−0.8–0.1)

BMI, body mass index; GV, growth velocity; %FFM, percentage of fat free mass measured by bioimpedance; REE, resting energy expenditure; PCDAI, pediatric Crohn’s disease activity index; PUCAI, pediatric ulcerative colitis activity index; ESR, erythrocyte sedimentation rate; CRP, C-reactive protein; IGF-1, insulin-like growth factor-1; IGFBP-3, insulin-like growth factor-binding protein 3. Data are reported as mean ± standard deviation (SD) (min–max values). ^a^ and ^b^ designate statistically significant differences (Student *t*-tests) between IBD groups and Healthy controls at baseline, as 95% and 99% confidence interval, respectively. ** CRP median values were 3.0, 2.0, 2.0 for CD at T0, T6 and T12, and 5.0, 2.0, 3.0 for UC at T0, T6 and T12, respectively. No statistically significant changes were observed in CD and UC groups over time. * One UC subject did not provide urine at baseline. NA: Not available.

**Table 2:** Urine metabolite patterns in the IBD subjects and healthy controls.

Group	Healthy Controls	CD			UC		
Metabolites (a.u.)/Visit	T0	T0	T6	T12	T0	T6	T12
Uk1	4.6 ± 2.5	4.1 ± 2.3	4.3 ± 2.0	3.2 ± 1.2 ^b^	3.7 ± 1.5	6.2 ± 3.2	7.9 ± 7.5 ^b^
Uk2	13.1 ± 1.2	13.5 ± 1.6	13.4 ± 1.9	14.2 ± 3.5	15.4 ± 3.2 ^a^	13.8 ± 1.5	16.4 ± 5.1 ^a^
Uk3	40.9 ± 4.6	59.7 ± 53.0 ^b^	50 ± 22.0 ^a^	96.7 ± 150.9 ^b^	184.4 ± 166.3 ^a^	90 ± 83.8 ^a^	263.9 ± 259.6 ^a^
Methanol	37.4 ± 8.7	36.0 ± 12.3	30.2 ± 6.6 ^a^	33.9 ± 9.3	35.0 ± 4.0	35.5 ± 14.3	39.7 ± 17.7
Acyl-carnitine	95.7 ± 15.6	99.3 ± 22.1	108.6 ± 30.6 ^b^	99 ± 27.4	93.6 ± 18.3	81.9 ± 16.6 ^b^	82.9 ± 12.9 ^b^
*cis*-Aconitate	36.4 ± 4.6	31.1 ± 7.7 ^a^	30.2 ± 6.9 ^a^	30.1 ± 5.6 ^a^	30.2 ± 1.7 ^a^	29.2 ± 3.0 ^a^	27.9 ± 4.7 ^a^
Betaine	179.9 ± 62.3	215.2 ± 228.6	176.8 ± 86.9	163.1 ± 55.6	202.7 ± 53.3	144.5 ± 68.5	165.5 ± 78.9
Urea	380.0 ± 124.8	281.3 ± 153 ^a^	227.4 ± 139.9 ^a^	258.5 ± 147.7 ^a^	357.9 ± 89.6	289.1 ± 139.1	273.1 ± 142.5 ^b^
4-Hydroxyphenylacetate	3.4 ± 0.6	3.4 ± 0.7	3.1 ± 0.9	10.5 ± 20.1 ^b^	11.8 ± 14.2 ^a^	7.3 ± 6.6 ^a^	24.9 ± 28.7 ^a^
4-Hydroxyphenylpyruvate	13.3 ± 6.0	12.8 ± 4.8	9.6 ± 3.9 ^b^	20.8 ± 22.5	31.2 ± 26.5 ^a^	14.1 ± 10.3	35.2 ± 28.6 ^a^
Phenylacetylglutamine	6.9 ± 1.1	9.0 ± 4.5 ^a^	7.5 ± 1.2 ^b^	9.6 ± 3.8 ^a^	8.9 ± 1.6 ^a^	13.1 ± 12.4 ^a^	9.6 ± 2.5 ^a^
Tryptophan	6.6 ± 2.6	8.2 ± 7.0	7.4 ± 10.1	17.9 ± 43.9	49.2 ± 51.7 ^a^	20.3 ± 29.0 ^a^	68.4 ± 74.9 ^a^
Hippurate	140.9 ± 92.3	53.6 ± 32.8 ^a^	57.3 ± 45.6 ^a^	81.5 ± 69.6 ^b^	67.1 ± 32.1 ^b^	62.8 ± 59.6 ^b^	64.4 ± 50.2 ^b^
Glycine	91.7 ± 32.2	102 ± 39.4	103.5 ± 35.4	119.7 ± 68 ^b^	106.1 ± 38.9	128.6 ± 59 ^a^	122.9 ± 71.3
Taurine	117.2 ± 28.7	99.9 ± 37.0	118.1 ± 33.4	94.8 ± 37.1 ^a^	101.8 ± 35.9	81.8 ± 30 ^a^	112.7 ± 68
Mannitol	364.7 ± 67.9	404.5 ± 77.9 ^b^	379.7 ± 44.5	369 ± 30.4	379.4 ± 28.6	391.1 ± 95.2	351.7 ± 79
Carnitine	57.1 ± 31.3	35.4 ± 15.7 ^a^	51.4 ± 47.0	27.6 ± 7.9 ^a^	44.2 ± 27.6	41.8 ± 13.8	30.4 ± 12.6 ^b^
Succinate	27.2 ± 4.9	24.6 ± 5.9	24.6 ± 4.8	26.3 ± 6.4	22.1 ± 3.8 ^a^	28.8 ± 9.5	23.9 ± 3.7
3-Methyl-2-oxovalerate	16.1 ± 1.6	16.9 ± 2.6	17.4 ± 3.4	15.9 ± 2.1	15.5 ± 1.5	35.6 ± 46.9 ^a^	20.3 ± 11 ^a^
3-Hydroxyisobutyrate	15.0 ± 2.5	13.2 ± 3.2 ^a^	13.9 ± 3.9	13.4 ± 3.4	12.6 ± 1.8 ^a^	35.9 ± 55.4 ^a^	17.4 ± 9.4
2-Oxoisocaproate	21.7 ± 3.3	22.3 ± 3.9	22.5 ± 2.4	21.0 ± 3.4	20.1 ± 3.5	31.0 ± 20.0 ^a^	26.5 ± 9.6 ^a^
Citrate	180.9 ± 54.3	168 ± 79.2	178.1 ± 65.4	180.9 ± 85.0	152.3 ± 31.1	197.4 ± 156	196.5 ± 58
Creatinine	862.7 ± 152.6	866.3 ± 152.1	856.9 ± 152.6	844.7 ± 107.4	832 ± 101.7	841.0 ± 263.3	846.6 ± 140.5
3-aminoisobutyrate	17.9 ± 2.6	17.3 ± 3.2	18.9 ± 4.9	17.9 ± 4.3	18.2 ± 2.5	20.9 ± 9.4	18.4 ± 2.6
Lactate	49.9 ± 12.5	52.3 ± 8.9	156.4 ± 372.2	51.2 ± 12.1	47.9 ± 6.1	69.8 ± 50.5 ^b^	76.6 ± 67.6 ^b^
Formate	5.7 ± 1.8	4.6 ± 2.4	4.5 ± 2.7	4 ± 1.7 ^a^	4.6 ± 1.4	17.7 ± 30.4 ^a^	4.6 ± 1.7

Data are reported as mean ± SD. Relative quantitation is obtained through calculating the area under the curve for a representative signal in the nuclear magnetic resonance spectra, and are reported using an arbitrary unit (a.u.). ^a^ and ^b^, difference between IBD groups and healthy subjects are significant at 95% and 99% confidence interval, respectively. Uk 1, 2, 3: unassigned metabolite 1, 2 or 3.

**Table 3:** Clinical phenotypes.

Clinical Parameters	Group 1 (6% UC)	Group 2 (53% UC)	Group 3 (30% UC)	Healthy	*p*-Values
Weight z-score	−1.5 ± 0.5	0.4 ± 0.6	−0.7 ± 0.7	0.5 ± 1	^x,y,z,a,c^
Height z-score	−0.8 ± 0.4	-0.2 ± 1.2	−0.7 ± 1.3	0.5 ± 0.9	^x,a,b,c^
BMI z-score	−1.6 ± 0.7	0.5 ± 0.8	−0.4 ± 0.7	0.4 ± 0.9	^x,y,z,a,c^
GV z-score	−0.2 ± 0.9	0.4 ± 1.5	1.2 ± 1.8	NA	^z^
%FFM (kg on the weight)	32.3 ± 3.7	38.1 ± 4	39.7 ± 6.3	39.8 ± 11.3	^x,z,a^
REE (kcal)	1284.9 ± 128.6	1452.9 ± 152.9	1401.6 ± 216.4	1537.1 ± 284.8	^x,a^
Blood urea (mmol/L)	381.7 ± 128.9	321.1 ± 133.6	442 ± 90.4	437.9 ± 114.1	^y,b^
Caloric intake (Kcal/day)	2094.1 ± 422.5	1504.3 ± 256.5	1834 ± 581.6	1935 ± 461.1	^x,y,b^
ESR (mm/h)	21.6 ± 19.2	32.2 ± 26.3	9.8 ± 5.5	NA	^y,z^
CRP (mg/L)	11.6 ± 16.9	8.4 ± 9.3	2.7 ± 1.4	NA	^y,z^
Fecal calprotectin (µg/g)	689.2 ± 614.7	1086.4 ± 598.1	516.7 ± 635.2	NA	^y^
IGF-1 z-score	−0.7 ± 0.2	−0.4 ± 0.5	−0.6 ± 0.3	NA	^x^
IGFBP-3 z-score	−0.5 ± 0.2	−0.4 ± 0.3	−0.5 ± 0.2	NA	^x^

BMI, body mass index; GV, growth velocity; %FFM, percentage of fat free mass measured by bioimpedance; REE, resting energy expenditure. Data are reported as mean ± SD. ^x,y,z^, difference between groups 1∔2, 2∔3, and 1∔3 is statistically significant at 95% confidence interval, respectively. ^a,b,c^ difference between Healthy-group 1, Healthy-group 2 and Healthy-group 3 is statistically significant at 95% confidence interval, respectively.

**Table 4:** Urinary metabolites and clinical phenotypes.

Metabolites (a.u.)	Group 1	Group 2	Group 3	Healthy Controls	*p*-Values
Uk3	44.7 ± 5.6	142.7 ± 186.2	108.8 ± 131.2	40.9 ± 4.6	^x,a,b,c^
Methanol	36.5 ± 11.9	39.9 ± 12.8	29.4 ± 5.6	37.4 ± 8.7	^y,z,c^
Acyl-carnitine	115 ± 26.7	90.6 ± 16	90.2 ± 23.5	95.7 ± 15.6	^x,z,a^
*cis*-Aconitate	31.5 ± 5.2	30.3 ± 7.5	28.9 ± 5.1	36.4 ± 4.6	^a,b,c^
Urea	295.2 ± 157.1	308.1 ± 111.7	228.4 ± 140.6	380 ± 124.8	^c^
4-Hydroxyphenylacetate	4.3 ± 3.6	11.7 ± 19.6	8.5 ± 15.2	3.4 ± 0.6	^b^
4-Hydroxyphenylpyruvate	10 ± 5.8	24 ± 23.9	18.8 ± 16.6	13.3 ± 6	^x,z,b^
Phenylacetylglutamine	7.8 ± 2.2	11.5 ± 8	8.7 ± 2.7	6.9 ± 1.1	^b,c^
Tryptophane	5.1 ± 2.2	32.5 ± 54.2	24.1 ± 39	6.6 ± 2.6	^b,c^
Hippurate	57.9 ± 37.2	55.2 ± 60.1	73.4 ± 47.2	140.9 ± 92.3	^a,b,c^
Glycine	97.7 ± 31.5	108 ± 43.2	123 ± 62.9	91.7 ± 32.2	^c^
Taurine	106.8 ± 33.7	95.3 ± 38.8	104.9 ± 44.3	117.2 ± 28.7	^b^
Mannitol	381.7 ± 53.7	411.5 ± 67.7	361.8 ± 56.5	364.7 ± 67.9	^y,b^
Carnitine	38.1 ± 21.2	36.2 ± 11.9	39.9 ± 36.5	57.1 ± 31.3	^a,b^
Succinate	28.2 ± 5.9	23.6 ± 7	24.2 ± 4.4	27.2 ± 4.9	^z,c^
Creatinine	785.6 ± 107.6	826.1 ± 179.3	914.3 ± 125.3	862.7 ± 152.6	^z^

Data are reported as mean ± SD. Relative quantitation is obtained through calculating the area under the curve for a representative signal in the nuclear magnetic resonance spectra, and are reported using an arbitrary unit (a.u.). ^x,y,z^, difference between groups 1—2, 2—3 and 1—3 is statistically significant at 95% confidence interval, respectively. ^a,b,c^ difference between Healthy-group 1, Healthy-group 2, and Healthy-group 3 is statistically significant at 95% confidence interval, respectively. Legend: Uk3: unassigned metabolite 3.

## Supplementary Materials

Supplementary materials can be found at www.mdpi.com/1422-0067/17/8/1310/s1.
