# Supplementary material for: Urinary Metabolic Phenotyping Reveals Differences in the Metabolic Status of Healthy and Inflammatory Bowel Disease (IBD) Children in Relation to Growth and Disease Activity
Source: Int J Mol Sci. 2016 Aug 11;17(8):1310. doi: 10.3390/ijms17081310 (PMC5000707; doi:10.3390/ijms17081310)
Supplement: Supplementary file 1 [file ijms-17-01310-s001.pdf]

Supplementary Materials: Urinary Metabolic Phenotyping Reveals Differences in the Metabolic Status of Healthy and Inflammatory Bowel Disease (IBD) children in Relation to Growth and Disease Activity

Francois-Pierre Martin, Jessica Ezri, Ornella Cominetti, Laeticia Da Silva, Martin Kussmann, Jean-Philippe Godin and Andreas Nydegger

1. Supplementary Results

Variables identified by multivariate analysis were further probed by univariate testing as indicated in Table 2. When compared to healthy subjects, pediatric IBD patients show higher urinary excretion of phenylacetylglutamine (PAG), plus an unassigned metabolite giving resonance at ^{1}H 2.17 ppm (Uk3, Table 2); and lower urinary excretion of *cis*-aconitate, hippurate, and urea. Additional inspection of the UC subject spectra showed a consistently higher urinary excretion of acyl-carnitine, 4-hydroxyphenylacetate, tryptophan, α-keto-beta-methyl-*N*-valerate, 2-oxoisocaproate, and lactate when compared to healthy subjects. By contrast, CD subjects showed a lower excretion of carnitine when compared to healthy subjects.

2. Supplementary Discussion

UC patients show a consistent trend towards higher levels of other gut microbial metabolites, 4-hydroxyphenylacetate and 4-hydroxyphenypyruvate, that are mainly formed in the colon by bacterial fermentation [41,43], which may support region-specificity of gut metabolic dysbiosis.

Patients with UC have a consistent trend in higher urinary excretion of two products of branched chain amino acid (BCAA) metabolism, 3-methyl-2-oxovalerate and 2-oxoisocaproate, and lactate-end products of anaerobic carbohydrate metabolism, suggesting an upregulation of BCAA and carbohydrate catabolism. Concomitantly, urinary excretion of fatty acid β-oxidation intermediates, carnitine and acylcarnitine tends to decrease, thus indicating a downregulation of fatty acid breakdown through β-oxidation. Taken together with changes in PAG and the Krebs cycle, this urinary pattern describes a further remodeling of energy, amino acid and fatty acid metabolism in relation to the altered metabolic requirements of UC pediatric patients.

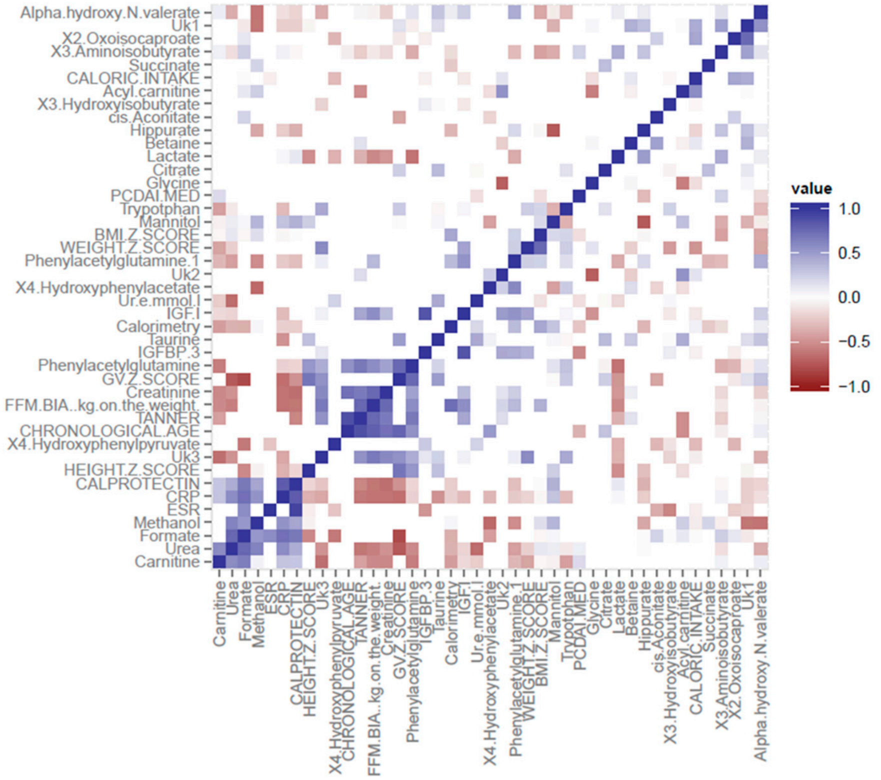

Figure S1. Correlation analysis between clinical and urine metabonome in CD children. Spearman correlation coefficient values shown are only those which are significant (after controlling for false discovery rate at a significance threshold of $\alpha = 0.01$). Blue represents a perfect correlation of 1, while red indicates a perfect anti-correlation, of −1.

Table S1. Therapeutic management of IBD patients.

Subject	Visit	Disease	Treatment					
			Steroids	5-ASA	Immunosuppresseurs	Biologicals	IPP	Calcium
2A-1	T0	CD				Yes	Yes	Yes
2A-1	T6	CD				Yes	Yes	Yes
2A-1	T12	CD				Yes	Yes	Yes
2A-10	T0	CD				Yes		Yes
2A-10	T6	CD				Yes		Yes
2A-10	T12	CD				Yes		Yes
2A-11	T0	CD			Yes			
2A-11	T6	CD			Yes			
2A-11	T12	CD			Yes			
2A-12	T0	UC		Yes				
2A-12	T6	UC		Yes				
2A-12	T12	UC		Yes				
2A-13	T0	UC		Yes	Yes	Yes		Yes
2A-13	T6	UC		Yes	Yes	Yes		Yes
2A-13	T12	UC		Yes	Yes	Yes		Yes
2A-14	T0	CD		Yes		Yes		Yes
2A-14	T6	CD		Yes		Yes		
2A-14	T12	CD		Yes		Yes		Yes
2A-15	T0	CD			Yes			
2A-15	T6	CD			Yes			
2A-15	T12	CD			Yes			

Table S1. *Cont.*

Subject	Visit	Disease	Treatment					
			**Steroids**	**5-ASA**	**Immunosuppresseurs**	**Biologicals**	**IPP**	**Calcium**
2A-16	T0	CD			Yes			Yes
2A-17	T6	UC				Yes		Yes
2A-17	T12	UC				Yes		Yes
2A-2	T0	CD				Yes		
2A-2	T6	CD				Yes		
2A-2	T12	CD			Yes			
2A-3	T0	CD			Yes			
2A-3	T6	CD			Yes			
2A-3	T12	CD			Yes			
2A-4	T0	CD				Yes		
2A-4	T6	CD				Yes		
2A-4	T12	CD				Yes		
2A-5	T0	UC				Yes		
2A-5	T6	UC				Yes		
2A-5	T12	UC				Yes		
2A-6	T0	CD				Yes		
2A-6	T6	CD				Yes		
2A-7	T0	CD			Yes			
2A-7	T6	CD			Yes			
2A-7	T12	CD			Yes			
2A-8	T0	CD			Yes			
2A-8	T6	CD				Yes		Yes
2A-8	T12	CD				Yes		Yes
2A-9	T0	UC		Yes		Yes		
2A-9	T6	UC		Yes		Yes		
2A-9	T12	UC		Yes		Yes		
2B-1	T0	CD	Yes			Yes		Yes
2B-1	T6	CD				Yes		Yes
2B-1	T12	CD			Yes	Yes		Yes
2B-2	T0	CD	Yes		Yes		Yes	Yes
2B-2	T6	CD				Yes		
2B-2	T12	CD				Yes		
2B-3	T0	UC		Yes		Yes		
2B-3	T6	UC		Yes		Yes		
2B-3	T12	UC		Yes	Yes	Yes		
2B-4	T0	CD	Yes	Yes			Yes	Yes
2B-4	T6	CD			Yes			
